# Optimising acquisition parameters for myocardial T2 mapping using T2-prep at 3T

**DOI:** 10.1186/1532-429X-15-S1-W10

**Published:** 2013-01-30

**Authors:** EM Tunnicliffe, MD Robson

**Affiliations:** 1 OCMR, Radcliffe Department of Medicine, University of Oxford, Oxford, UK; 2 AVIC, Radcliffe Department of Medicine, University of Oxford, Oxford, UK

## Background

T2 mapping using T2-prepared SSFP at 1.5T has been shown to be sensitive to oedema in acute myocardial infarction[1]. At 3T, other studies have addressed the problem of the T2 prep module's sensitivity to the increased B1 inhomogeneity[2]. However, a second problem is that the lengthened T1 at 3T can reduce the contrast between normal and oedematous myocardium, as well as introducing heart rate variability in measured T2. We set out to develop a protocol which maximised the difference between measured T2 in normal and oedematous tissue, while minimising the heart-rate dependence of the measured T2.

## Methods

Two gel phantoms (agarose and NiCl_2_) were constructed with relaxation times close to normal myocardium (N) and oedematous myocardium (O)[1][3]. These relaxation times were measured at 3T (Siemens Verio) using ShMOLLI[3] and a multi-echo spin echo sequence.

Next, a T2-prepared T2 mapping sequence (Siemens WIP 447) was used, with the default 1.5T protocol[1] varied as follows: GRE readout, flip angle=5°, 9°,18°; SSFP readout, flip angle=20°,35°,50°; Linear and centric k-space ordering, and with the order of the T2 prep module echo times permuted (0,32,55ms;0,55,32ms etc.). Each protocol variant was run with heart periods of 600ms, 1000ms and 1200ms (heart rates of 50, 60 and 100 bpm), for a total of 216 scans.

The reconstructed T2 maps were analysed using Matlab (Natick, MA). Each protocol was evaluated for its ability to distinguish between normal and oedematous myocardium by comparing the difference between the measured T2, averaged over a ROI covering the body of the N and O phantoms. The standard deviation of measured T2 for each protocol across the three heart rates was used as a measure of heart-rate dependence. A t-test was used to determine whether the difference in T2 was statistically significant over all heart rates, and to rank the protocols, with the lowest p-value protocol providing the best discrimination between the T2 of the two phantoms.

## Results

**Figure 1:**
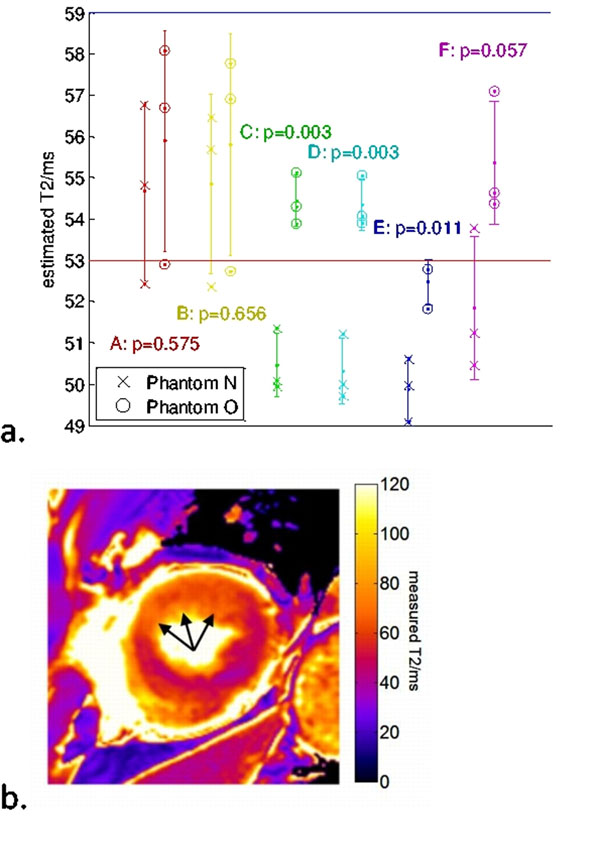
a. Measured T2 on phantoms with relaxation times close to normal (N) and oedematous (O) myocardium with a subset of the protocols tested. Red and blue dotted lines show the true T2 of phantoms N and O respectively. The three points for each phantom and protocol represent the three heart rates (with higher heart rates leading to longer measured T2 values) and error bars show the mean±s.d. over heart rate. Protocol details are in Table 1. b. Discrimination of oedematous (arrowed) and remote myocardium in acute myocardial infarction using protocol D.

The measured reference relaxation times for the two phantoms were N: T1/T2=1152/53ms; O: T1/T2=1302/59ms. Apparently minor changes in the acquisition protocol yield wildly different T2 values using these methods (Figure 1a). No protocol provided measurements of T2 within 3ms for both phantoms over all heart rates and many approaches demonstrated considerable bias from the true T2 value.

**Table 1:** Protocols for comparison are: the default 1.5T protocol, and default 1.5T protocol with GRE readout (A&B); the three best protocols (lowest p-value, i.e. best discrimination between measured T2), two GRE (C&D) and one SSFP (E); the best protocol with an SSFP readout substituted for GRE (F).

Protocol (Figure 1a)	Protocol details	Mean difference in T2/ms	Quadrature-combined s.d. over all heart rates	t-test p-value	Comment
A	SSFP, 50°, linear, 0-32-55ms	1.2	3.5	0.575	1.5T default protocol[1]

B	GRE, 9°, linear, 0-32-55ms	1.0	3.5	0.656	1.5T default protocol with GRE readout

C	GRE, 18°, centric, 32-0-55ms	4.0	1.0	0.003	Lowest p-value

D	GRE, 18°, centric, 32-55-0ms	4.0	1.0	0.003	Lowest p-value

E	SSFP, 20°, centric, 0-55-32ms	2.6	0.95	0.011	Lowest p-value with SSFP readout

F	SSFP, 50°, centric, 32-0-55ms	3.5	2.3	0.057	Best protocol but with SSFP readout

Selected pertinent protocol details and numeric results are summarised in Table 1. Figure 1b shows the detection of oedema in a patient using protocol D.

## Conclusions

The sensitivity of myocardial T2 mapping at 3T can be significantly improved by optimising acquisition parameters. Based on this phantom study, we use a centrically ordered GRE readout with a flip angle of 18° and a T2-prep order of 32-55-0ms.

## Funding

We thank the NIHR Oxford Biomedical Research Centre and UK Department of Health for grant funding.
